# Exploration in 4‐year‐old children is guided by learning progress and novelty

**DOI:** 10.1111/cdev.14158

**Published:** 2024-09-02

**Authors:** Francesco Poli, Marlene Meyer, Rogier B. Mars, Sabine Hunnius

**Affiliations:** ^1^ Donders Institute for Brain, Cognition and Behaviour Radboud University Nijmegen The Netherlands; ^2^ MRC Cognition and Brain Sciences Unit University of Cambridge Cambridge United Kingdom; ^3^ Nuffield Department of Clinical Neurosciences, Wellcome Centre for Integrative Neuroimaging, Centre for Functional MRI of the Brain (FMRIB), John Radcliffe Hospital University of Oxford Oxford UK

## Abstract

Humans are driven by an intrinsic motivation to learn, but the developmental origins of curiosity‐driven exploration remain unclear. We investigated the computational principles guiding 4‐year‐old children's exploration during a touchscreen game (*N* = 102, F = 49, M = 53, primarily white and middle‐class, data collected in the Netherlands from 2021–2023). Children guessed the location of characters that were hiding following predictable (yet noisy) patterns. Children could freely switch characters, which allowed us to quantify *when* they decided to explore something different and *what* they chose to explore. Bayesian modeling of their responses revealed that children selected activities that were more novel and offered greater learning progress (LP). Moreover, children's interest in making LP correlated with better learning performance. These findings highlight the importance of novelty and LP in guiding children's exploration.

**Abbreviations**
LP: learning progress
PE: prediction error

## INTRODUCTION

As adults, we rely on complex knowledge to optimally navigate our physical and social world. However, children need to acquire this knowledge from scratch. Especially during preschool years, a large part of their learning depends on their own abilities to ask questions (Ruggeri et al., 2017), seek information (Hembacher et al., 2020; Sehl et al., 2021), and actively explore (Meder et al., 2021), which allow them to constantly improve their knowledge of the world. Remarkably enough, between 3 and 4 years of age, children ask more than 100 information‐seeking questions per hour (Chouinard, 2007). Children's exploration might thus be curiosity driven (Oudeyer et al., 2016; Poli, O'Reilly, et al., 2024), in that acquiring information is intrinsically rewarding by itself. Yet, the computational principles that drive preschoolers' exploration behavior are still unknown.

Previous research has studied *what* children's exploration skills are: Preschoolers explore more in response to unexpected events (Schulz et al., 2008), and their exploration is specifically directed at identifying an explanation for such events (Schulz & Sommerville, 2006; Sobel et al., 2007). When multiple hypotheses can explain the same event, preschoolers' exploration is aimed at dissociating them (Cook et al., 2011; van Schijndel et al., 2015), and the less discernible competing hypotheses are, the more the preschoolers explore (Siegel et al., 2021). Their exploratory efforts are also flexible as children can adapt their exploration strategies to the task at hand (Ruggeri et al., 2019; Poli, Li, et al., 2024). Overall, this evidence indicates that children's exploration is both strategic and aimed at identifying explanations for surprising events.

We know less, however, about the principles that guide *how* children explore. Children might explore to minimize *surprising events* (Ronfard et al., 2021; Stahl & Feigenson, 2017; Stahl & Woods, 2022), where surprise originates from a mismatch between their expectations (i.e., predictions) about the world and the reality of a situation. For example, children might expect that objects fall to the ground when dropped. When they see a balloon flying up into the air instead of falling down, they experience a prediction error (PE). This PE may generate surprise, and the subsequent need to reduce it. This is consistent with the idea that children explore as long as their expectations are violated and stop when they find an explanation that allows them to adjust their expectations and reduce the surprise (Siegel et al., 2021). However, this specific strategy is not always efficient, as sometimes PEs cannot be reduced, either because some challenges are too difficult or because some aspects of the world are simply unlearnable (Gottlieb & Oudeyer, 2018; Poli, O'Reilly, et al., 2024).

A more efficient exploration strategy resides in seeking *learning progress* (LP) rather than minimizing surprise. This would lead to a focus on a certain activity only as long as it is possible to get better at it or to gain more knowledge on it, thereby maximizing learning (Andersen et al., 2022; Oudeyer et al., 2007). This exploration strategy allows an agent to avoid activities that are either too easy, where PEs have already been reduced and there is nothing left to learn, or too difficult, where PEs cannot be reduced and learning is impossible. Recent findings showed that adults' curiosity‐driven exploration follows this strategy (Poli et al., 2022). Specifically, Poli et al. (2022) found that when participants were learning more, they were more likely to stick with the same activity Conversely, they were more likely to explore a different activity when learning less. Moreover, when they had to decide what to explore next, adults chose activities from which they expected to learn the most.

In addition to LP, adults' exploration is also guided by the novelty of the available activities (Poli et al., 2022). *Seeking novelty*, irrespective of how useful it is for learning, is another factor that might account for children's exploratory behavior. This exploratory strategy is computationally simpler because it only requires tracking the familiarity of a stimulus, rather than the PE associated with it or the change in PE over time (i.e., the LP).

In this paper, we formalize prior distinct theories of children's exploration through a Bayesian modeling approach, and we test which one best explains preschoolers' exploration. Specifically, we test whether children's exploratory behavior is governed by PEs, LP, novelty, or a combination of them. We let 4‐year‐old children play a hide‐and‐seek computer game on a touchscreen. Children could interact with three different characters that were hiding following probabilistic patterns. We manipulated the levels of noise and volatility of these patterns to generate variability in the experimental factors of interest, that is, PEs, LP, and novelty (Nassar et al., 2019; O'Reilly, 2013). We introduced noise by adding randomness to the specific patterns that the characters were following, thus creating irreducible uncertainty in where they will hide. For example, if a character was hiding in the center of the screen, it would not appear always in the same exact position, but it would have a random offset from the center that made its location not fully predictable. In addition, we introduced volatility by manipulating the amount of sudden changes in the patterns from time to time. For example, a character hiding in the center of the screen might suddenly move its hiding position to a corner of the screen, and remain around that corner for some time, before changing again to another position. Volatility allowed us to create different degrees of reducible uncertainty in where the characters would hide. Then, we used a hierarchical reinforcement learning model (Nassar et al., 2010; Wittmann et al., 2016) to infer trial‐by‐trial estimates of children's PEs and LP from their performance on the task.

This novel task offers a way to probe children's curiosity‐driven exploration. The design integrates aspects of probabilistic learning tasks (Nassar et al., 2010; O'Reilly, 2013), in which participants are required to learn the latent structure of the task to obtain rewards, and patch‐foraging tasks (Constantino & Daw, 2015; Kolling et al., 2012), in which participants are asked to strategically explore different environments to maximize rewards. Crucially, our task includes both a probabilistic learning aspect, thus allowing children to improve their performance by trial and error, and a patch‐foraging aspect, which allows children to freely explore the different options that are available to them. The patch‐foraging aspect of the task offers a parsimonious way of probing whether at any moment in time, children prefer to exploit (i.e., stay focused on the same character) or explore (i.e., switch character) to investigate what drives their exploratory decisions. Crucially, our approach abstains from using extrinsic rewards to assess the balance between explorative and exploitative behaviors, a strategy that contrasts with traditional probabilistic learning and patch‐foraging tasks (Wilson et al., 2021). This absence of external motivators positions our study to offer valuable insights into the intrinsic motivations that underpin children's exploration decisions, potentially highlighting the curiosity inherent in early childhood.

## METHODS

### Participants

A total of 102 children (*M*_age_ = 47.37 months, SD = 16 days, range = 46–48 months, 49 females, 53 males) were recruited for the study from a database of volunteer families between September 2021 and January 2023 and were invited to the lab via phone calls. We targeted this age group because they have not entered formal schooling yet, but are old enough to fully understand verbal instructions and maintain enough focus on the task we designed. Children were primarily White and from middle‐class families, Dutch speaking, and living in a medium‐sized European city (Nijmegen, the Netherlands). The sample size was determined based on previous studies on adults (Poli et al., 2022; Poli, Koolen, et al., 2023), adjusted for the increased amount of noise that is expected in children's data.

One participant was discarded from the analysis because of parental interference during the task, and 15 additional participants were discarded because they did not meet the inclusion criteria of playing with all available characters for at least 10 trials. This criterion was chosen because the computational model fails to converge when data are too sparse. The final sample consisted of 87 children (*M*_age_ = 47.38 months, SD = 17 days, 42 females). Families received a children's book or €10 for their participation. The local ethics review board approved the study.

### Materials and procedure

**FIGURE 1:**
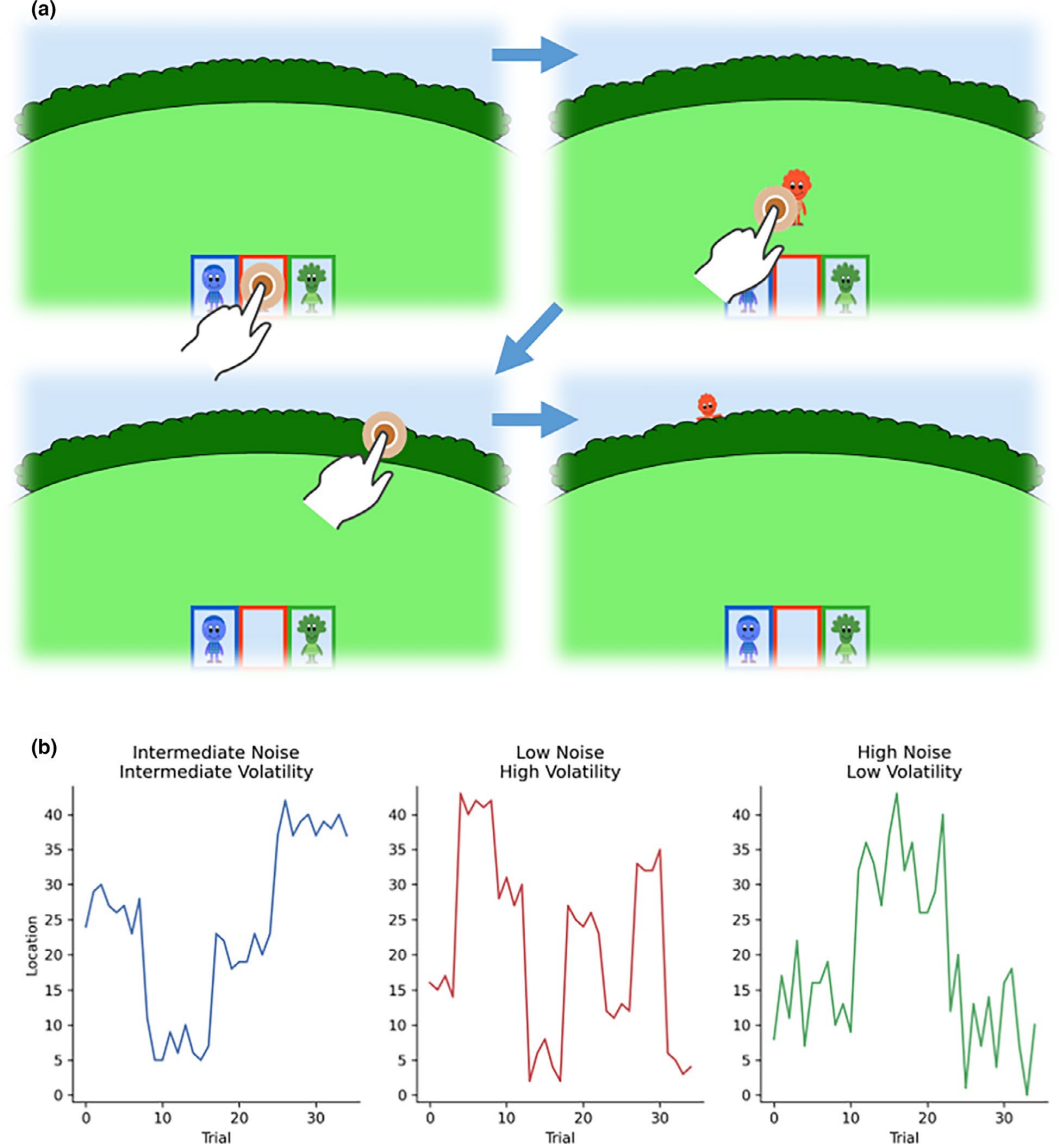
The Exploration Task. (a) Participants started the task by choosing which character to play with and had to click on it to make it hide behind the hedge. After they guessed where the character would reappear, the character appeared in its actual location. Then, a new trial started, with the character appearing in the middle of the screen again. Children could decide whether to keep playing with it or switch to a different character. (b) The hiding patterns of the three characters across time. On the *y*‐axis, 0 indicates the far‐left corner of the hedge. The hiding patterns featured different degrees of noise and volatility. The noise resulted in the character's hiding spot never being exactly the same. The volatility in the pattern made the hiding location change more or less frequently to a different spot.

The task was coded in PsychoPy 3.2 (Peirce et al., 2019) and uploaded on Pavlovia (pavlovia.org). Children played on a large touchscreen (22″) while the session was video recorded. Children were presented with three distinct characters (Figure 1). To start, the experimenter introduced the task by saying that three characters wanted to play hide and seek. Children were asked to decide which of the three characters they wanted to play with first by tapping on it. The selected character appeared in the middle of the screen, below a long hedge. Then, children were told that the character was ready to hide and to tap on it again to make it disappear. At this point, the experimenter asked where the character was hiding behind the bushes, and whether the child could tap where the character was. Participants had to touch the hedge, trying to guess where the character would reappear. Upon touching the screen, the character reappeared from behind the hedge.

After this introductory trial, the children were instructed that they could click on another character to switch characters and that this would allow them to play with all the characters. Finally, they were asked if they wanted to play again, and if so, which character they wanted to play with. No further instructions were given on where the characters would hide or how to find them. Also, no external rewards were given for correctly guessing the character's hiding spot. When children got distracted and stopped playing, the researcher would prompt them asking if they wanted to play more. The session would end when children played with any of the characters for at least 35 trials, or after they declined the researcher's invite to play for longer. Hence, participants differed in the overall number of trials played (*M* = 27.8, SD = 15.7) and in the time in minutes spent on the task (*M* = 4.21, SD = 1.64).

Across the task, two aspects of children's exploration behavior were recorded. First, we classified every trial as a stay trial if children kept playing with the same character, and as a leave trial if they switched characters. These are labeled as leave–stay decisions. Second, any time children switched the character with which they were playing, we recorded what character they switched to among the two options that were available. These are labeled as exploratory decisions.

The three characters were hiding following separate Gaussian distributions. Two parameters of the Gaussian distribution (mean and standard deviation) were manipulated independently for each character so that the three characters followed three different hiding patterns (Figure 1b): They had three different levels of noise (i.e., the standard deviation of the Gaussian distribution) and three different levels of volatility (i.e., how often the mean of the distribution changed). The first character had high noise and low volatility, the second had low noise and high volatility, and the third had intermediate noise and volatility. Which character (blue, green, or red) was paired with each hiding pattern was counterbalanced across participants.

### Analysis

#### General approach

**FIGURE 2:**
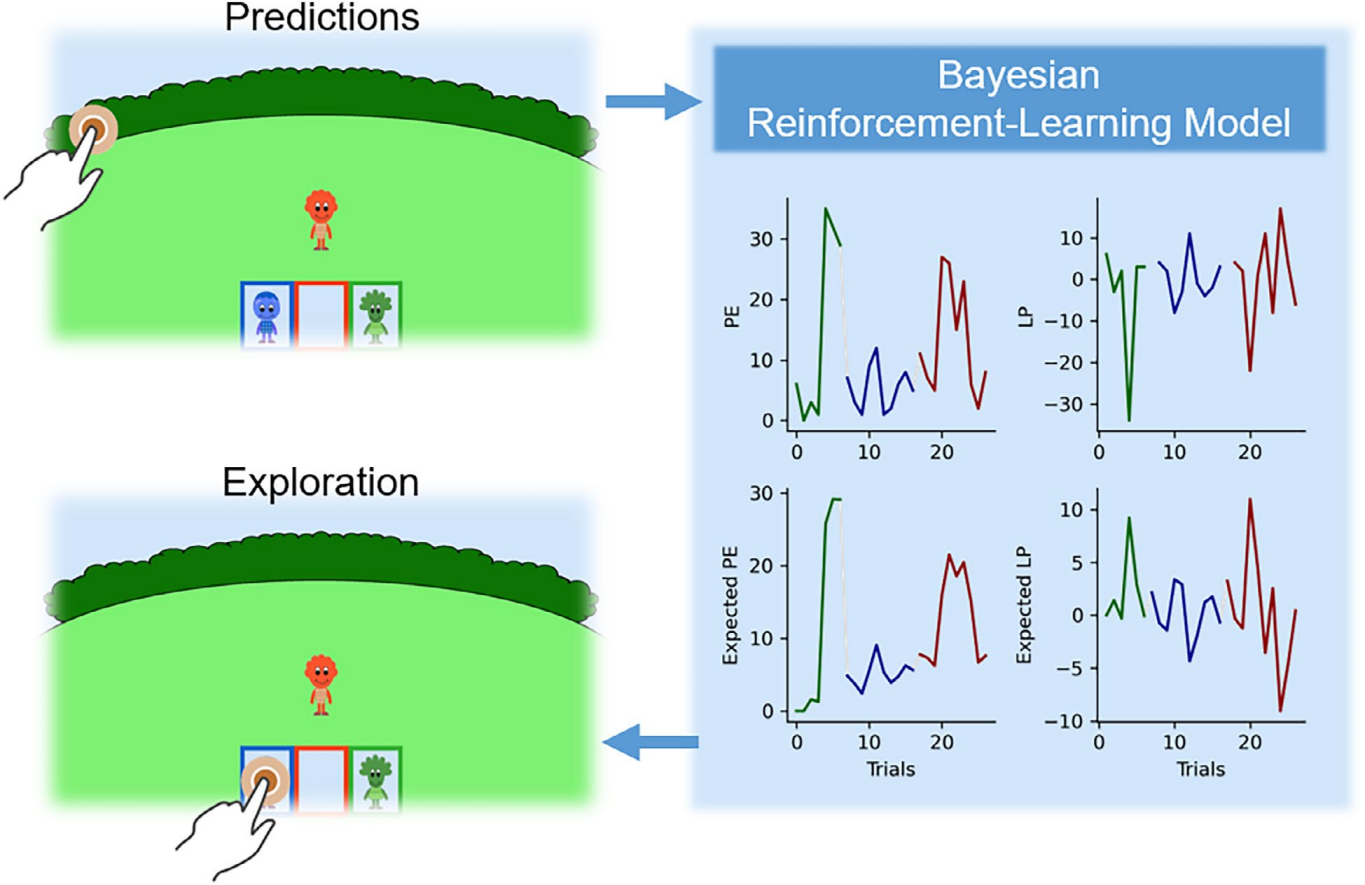
Computational modeling of behavior. We used children's trial‐by‐trial predictions of the character locations as input for the Bayesian reinforcement learning model. The model estimates (expected) prediction error (PE) and (expected) learning progress (LP). (Different colors indicate different characters.) These estimates were used to predict children's leave–stay decisions (i.e., choose a new character vs. stay with the same one) and exploratory decisions (in case a leave decision is made, what character is chosen next?).

The goal of our analyses was to predict how PE, LP, and novelty impacted children's exploration behavior. Specifically, these factors may affect decisions on whether to stop playing with a certain character (i.e., leave–stay decisions) and decisions on what to explore next (i.e., exploratory decisions). These factors were quantified using a Bayesian reinforcement learning model that we previously developed and fitted on adults' data (Poli et al., 2022). The model uses the trial‐by‐trial behavioral predictions of the character's hiding location made by the participants as input and estimates for each trial the PE and LP as well as its novelty (Figure 2). Once these trial‐by‐trial estimates were obtained, we related them to exploratory decisions and leave–stay decisions via generalized linear models. When results belong to a logistic regression, odds ratios are reported for confidence intervals. For these, the null hypothesis does not correspond to the value of 0, but 1. Hence, when 1 is included in the interval, the null hypothesis is not rejected. All analyses were confirmatory.

#### Quantifying PEs, LP, and novelty

**FIGURE 3:**
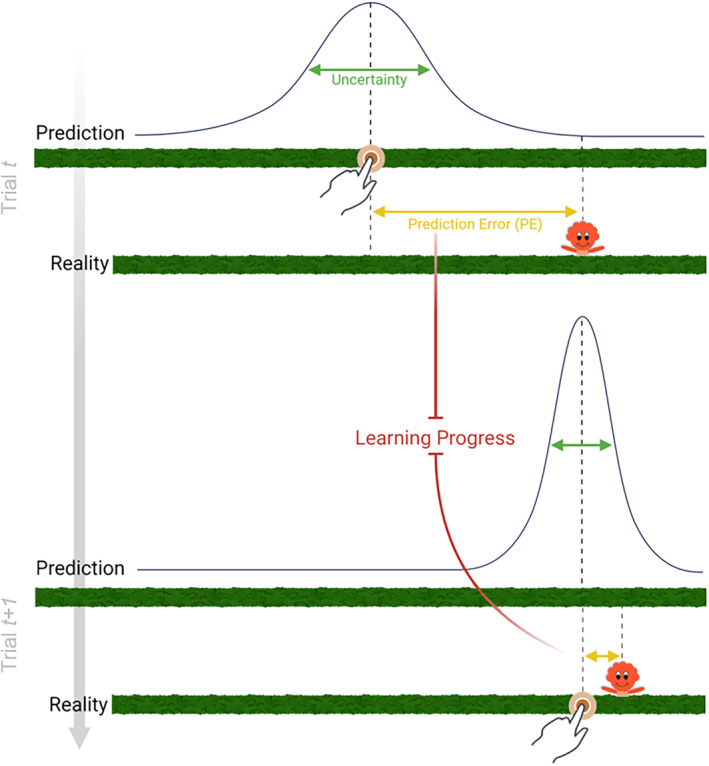
Examples of prediction errors (PEs) and learning progress (LP) in the task. On every trial, participants predicted the hiding location of the character (with some degree of uncertainty) and observed its actual location (reality). The difference between predicted and actual location is the PE. Tracking how the PE changes from one trial to the next allows to compute the LP. The Bayesian model also computes the PE and the LP that are expected in the future given the current learning rate.

On every trial, participants made a prediction about the character's location. Once the prediction was made, the character appeared, revealing its actual location. PE was computed as the difference between the location of the character that the participant predicted and the actual location of the character (Figure 3, in yellow). The greater the distance between predicted and actual location, the higher the PE. By tracking how PEs changed over trials, the model measured LP. Specifically, LP is the degree of change in PE over two consecutive trials (Figure 3, in red). For example, if a participant made a large PE in the first trial and a small PE in the second trial, this resulted in a big decrease in PE, thus indicating a high level of LP. Conversely, if the PE in the second trial remained high, this resulted in a low level of LP. LP can also be negative if PEs increase over time, which indicates a worsening of the performance. Finally, the degree of novelty is inferred by the number of times a character has been picked: The more a character has been interacted with, the less novel it is. This operationalization of novelty as “negative familiarity” is dissociable from PE both computationally and experimentally (for a review, see Modirshanechi et al., 2023).

The Bayesian model did not only estimate the *current* levels of PE and LP but also the PE and the LP that participants *expected* to make in the following trial. Examples of expected LP and PEs are reported in Figure 2. These measurements are crucial because when participants decide what character to engage with next, they rely on their expectations of how big LP or PEs will be in the future, rather than solely on past experiences (see Poli et al., 2022). The details of how they were computed are described in the Supporting Information.

## RESULTS

### Variability in children's learning

Before assessing what guides children's exploration behavior, we tested whether children learned the hiding patterns of the characters successfully over the course of the experiment. This was done by testing whether PEs decreased over trials (i.e., whether children got better over time). Specifically, we assessed whether PEs decreased across consecutive trials that were played with the same character. When the character changed, trial number would reset. If performance assessed in this way improves over trials, we can conclude that successful learning has occurred.

**FIGURE 4:**
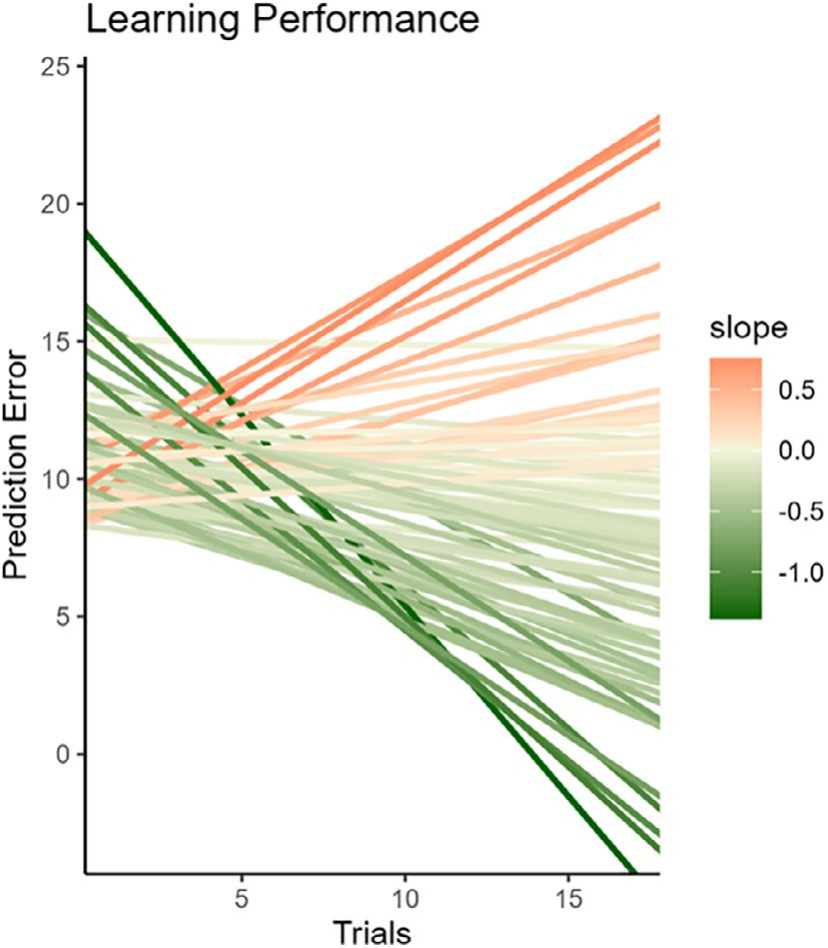
Individual differences in performance. Reduction of prediction errors over trials is considered an index of performance. Children display different slopes, which indicate different performance. The color gradient indicates the specific value of each child's performance.

At the group level, we did not find a significant improvement in performance over time, as indicated by the effect of time on the estimates of the PE (*t* = −.81, *β* = −.04, *p* = .42). Random effects reveal substantial individual differences (Figure 4), suggesting that some children learned successfully, while some did not learn. Further analyses showed that these differences in performance were not related to how often participants switched character (*t* = −.26, *β* = −.001, *p* = .80) or how long they played for (*t* = −1.20, *β* = −.001, *p* = .24). We included performance in all the following analyses, as we may expect different results depending on whether children have learned or not.

### Leave–stay decisions

**FIGURE 5:**
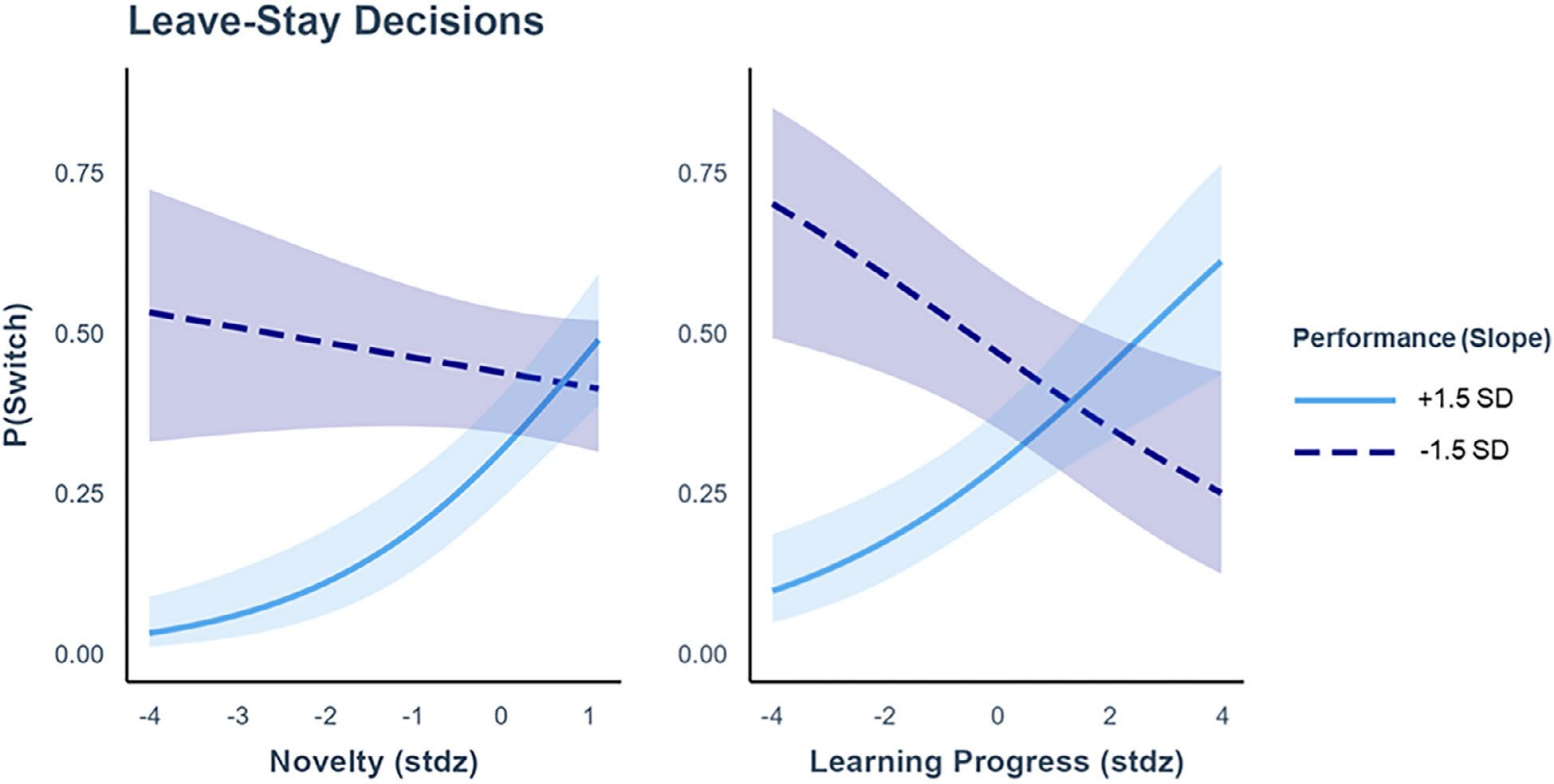
Leave–stay decisions depend on novelty and learning progress (LP) in interaction with performance. Children with better performance (i.e., negative slopes for prediction error over time) (dashed lines) were not affected by the novelty of the character when deciding whether to switch characters (left). However, they were more likely to switch characters when LP was lower (right). Children with worse performance (solid line) were less likely to stop playing with a character when it was more familiar (left) or associated with greater LP (right).

We examined whether children decided to stop engaging with a character based on their PEs, LP, or the novelty of the character (Figure 5). Specifically, we used a generalized linear model to predict whether, on each trial, participants stayed in the same environment or switched to a different one, while controlling for the main effect of time. The performance of each child (i.e., the slope in Figure 4) was included as interaction term. Participants were included as random effects in the model. We found a significant interaction of performance with novelty (*z* = 2.80, *β* = .73, *p* = .005, e^*β*^ = 2.08, 95% CI [1.24, 3.53]) and with LP (*z* = 2.76, *β* = .49, *p* = .006, e^*β*^ = 1.63, 95% CI [1.15, 2.33]), but no effect of PE (*z* = −0.15, *β* = −.01, *p* = .88, e^*β*^ = 0.99, 95% CI [0.86, 1.20]) and no effect of time (*z* = −1.05, *β* = −.12, *p* = .29, e^*β*^ = 0.89, 95% CI [0.70, 1.11]). This pattern of results indicates that leave–stay decisions depended on both the LP participants were making, and the novelty of the character they were playing with, but this effect changed depending on their performance.

To better understand how leave–stay decisions varied as a function of the children's performance, we estimated the marginal effects of novelty and LP. This enables us to compare how the effects of novelty and LP change across different levels of performance. This analysis showed that children with higher performance showed no effect of novelty (*β* = −.45, *p* = .075, 95% CI [−0.94, 0.04]) and were less likely to switch away as LP increased (*β* = −.51, *p* = .025, 95% CI [−0.95, −0.06]), while children with lower performance were less likely to switch away as novelty decreased (*β* = .96, *p* < .001, 95% CI [0.41, 1.52]) and as LP increased (*β* = .54, *p* = .004, 95% CI [0.18, 0.90]). These results are depicted in Figure 5. Since high correlations between independent variables can impact the validity and interpretation of the results, we checked for multicollinearity using the variance inflation factor. All variables had a factor <2.3, indicating low multicollinearity.

### Exploratory decisions

**FIGURE 6:**
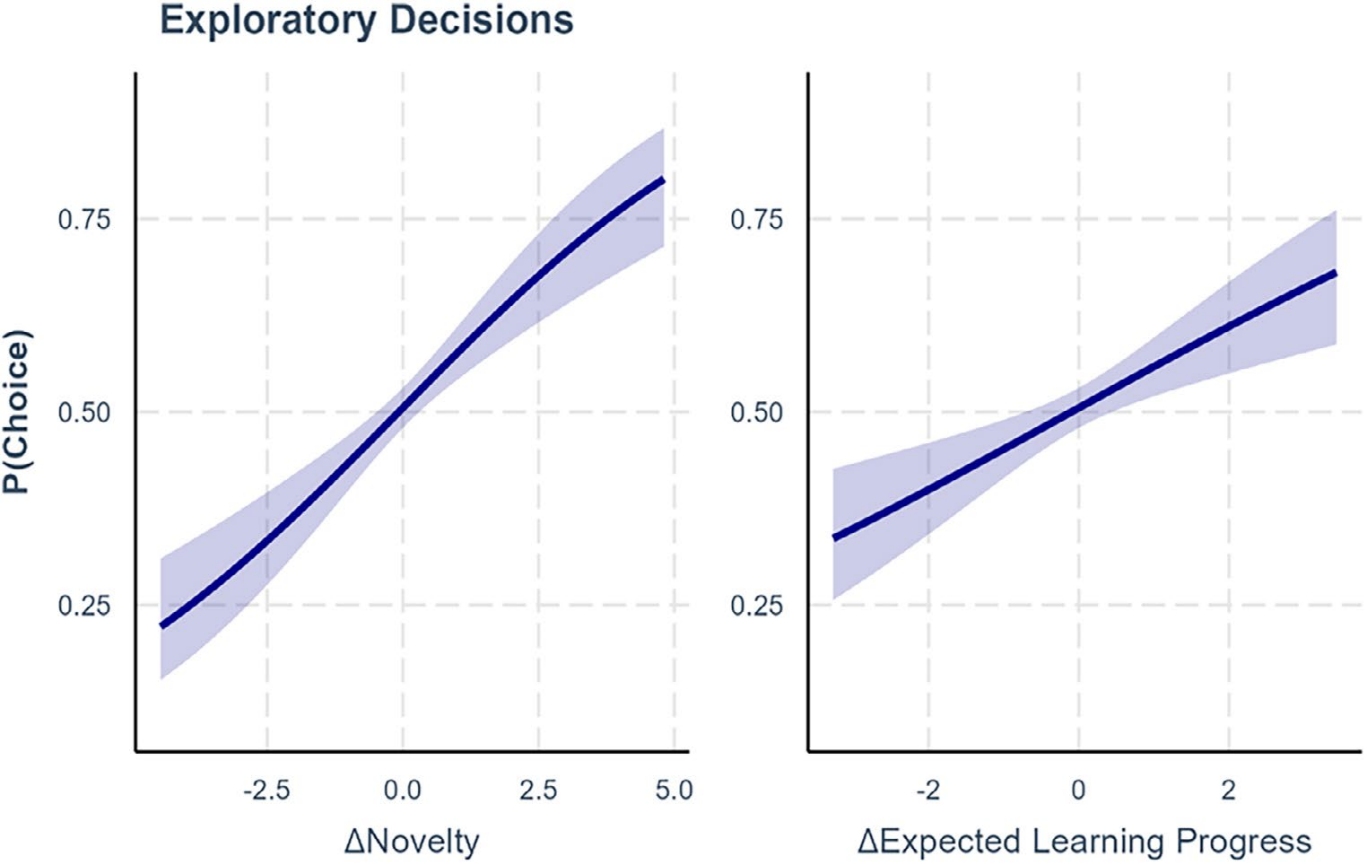
Exploratory decisions depend on novelty and expected learning progress (LP). Children were more likely to explore characters that were more novel (left) and that promised greater LP (right). The performance of the children did not influence their exploratory decisions.

We tested whether children decided what character to explore based on the PE or LP that could be expected, or on the novelty of the character (Figure 6). Since expectations can be computed only if participants had already interacted with the characters, the first switch to each character was excluded from analysis. We fitted a generalized linear model on binomial data with differential expected LP, differential expected PE, and differential novelty as predictors. Interaction terms for time and performance were added, and participants were included as random factor. The results show that both novelty (*z* = 3.82, *β* = .30, *p* < .001, e^*β*^ = 1.34, 95% CI [1.16, 1.58]) and expected LP (*z* = 2.03, *β* = .35, *p* = .04, e^*β*^ = 1.42, 95% CI [1.02, 1.99]) had a positive correlation with participants' choices. Conversely, expected PE did not have an effect (*z* = −1.18, *β* = −.21, *p* = .23, e^*β*^ = 0.81, 95% CI [0.56, 1.15]). We did not find any significant interaction between time and expected LP (*z* = −1.40, *β* = −.01, *p* = .16, e^*β*^ = 0.99, 95% CI [0.98, 1.00]), and between time and expected PE (*z* = 1.49, *β* = .01, *p* = .14, e^*β*^ = 1.01, 95% CI [0.99, 1.02]). Similarly, we did not find any interaction between performance and expected LP (*z* = −0.77, *β* = −.16, *p* = .44, e^*β*^ = 0.85, 95% CI [0.56, 1.28]), between performance and expected PE (*z* = 1.29, *β* = .34, *p* = .20, e^*β*^ = 1.22, 95% CI [0.84, 2.39]), and between performance and novelty (*z* = 1.49, *β* = .33, *p* = .14, e^*β*^ = 1.39, 95% CI [0.90, 2.14]). This pattern of results indicates that participants were more likely to pick characters from which they expected to learn more, as well as characters that were more novel, with no evidence that this pattern of results changes depending on the children's performance.

To substantiate the fact that children were relying on their expectations about LP and PE, rather than on previous performance, we compared the goodness of fit of the model reported above with a model with the same fixed and random effects, but replacing expected LP and expected PE with LP and PE (i.e., previous performance instead of expectations). The model relying on expectations performed better, as indexed by lower Akaike information criterion (714 vs. 721) and lower Bayesian information criterion (761 vs. 768).

## DISCUSSION

Curiosity‐driven exploration is crucial for early acquisition of knowledge and skills because it allows children to optimize their learning by directing their attention to specific, relevant aspects of the world. In this study, we investigated the principles that guide exploration in 4‐year‐old children. At this age, children are constantly asking questions and seeking information, but they rely on exploration strategies that do not yet depend on formal schooling. We examined these exploration strategies by presenting children with multiple activities and tracking what they decided to engage with and when. While children were exploring, we tracked their moment‐to‐moment changes in PE and LP, as well as the novelty of the stimuli. We related these estimates to their exploration behavior to determine what guides their curiosity.

We show that children decide what to explore not only depending on the novelty of the activity but also on how much they expect to learn from it. This is consistent with studies that reported advanced information‐seeking abilities in 4‐year‐olds when the task is more constrained (Ruggeri et al., 2019), and with research on older children and adults showing that individuals are not solely attracted by novel events, but possess the ability to preferentially explore the activities from which they can expect to learn the most (Liquin et al., 2021; Poli et al., 2022; Poli, Koolen, et al., 2023). Interestingly, previous research has shown that even infants engage with a stimulus only as long as it offers a learning opportunity (Poli et al., 2020). Together, these findings speak for some level of continuity in exploratory mechanisms across the lifespan.

Previous research showed that children look for explanations when their expectations are violated (Chu & Schulz, 2020; Stahl & Feigenson, 2017). Our findings contribute to explaining this phenomenon in two important ways. First, we found that exploration was directed toward activities that promised greater LP. In this perspective, the tendency to seek explanations can be framed as one specific strategy that would allow learning to proceed further. Second, theories of exploration as explanations do not make direct predictions of what happens when things are too difficult or impossible to explain. Conversely, the current study offers a mechanistic answer to how high‐performing children respond to unlearnable situations: LP offers a way to signal when to give up. Specifically, when PEs do not reduce and LP fails to occur, children are more likely to disengage.

We identified interindividual differences in performance, with some children showing clear signs of learning and others failing to show an improvement in their predictions over time. Crucially, these differences were related to children's exploration, in particular to their leave–stay decisions. Children with better performance were more likely to disengage from an activity when their LP was low, while we found no evidence that they were affected by the novelty of the stimuli. Conversely, children with poorer performance were more likely to stick to familiar activities and abandoned them if their LP was higher. It should be noted, however, that causality could go both ways. For example, it might be that children who remain focused on familiar activities might end up performing worse. An alternative interpretation is that children who are worse at the task end up focusing on familiar activities. Although these alternatives cannot be dissociated based on the current data, the interaction between performance and novelty (as well as between performance and LP) predicted individual differences in leave–stay decisions. This finding can be interpreted in relation to recent empirical evidence on children's exploration. A recent blicket detection study (Sobel et al., 2022) also identified differences in exploration within subgroups of children. Specifically, while exploring what elements would activate a machine, some children were more motivated to keep the machine activated, while other children were trying to identify cases where the machine failed to activate. In relation to this, it is possible that some children in our study were trying to improve their performance, while others were looking for instances that were violating what they already knew, and these different motivations may have contributed to the variability that we observed.

The individual differences in children's exploration strategies that we find may have important implications for pedagogical practices. In relation to the principle of personalized learning (Pane et al., 2015), children who excel by focusing on familiar activities might benefit from curricula that build progressively on prior knowledge. This incremental approach allows them to consolidate their understanding before branching out to novel topics. On the other hand, for those children driven by novelty, a wider curriculum offering a broad spectrum of subjects and resources might be the key to sustained engagement and effective learning. Moreover, our findings suggest that educators should make LP palpable to the students (Hmelo‐Silver et al., 2007). When children can discern their learning trajectory, their motivation to delve deeper into subjects may be enhanced. This implies a potential overhaul in assessment strategies, shifting from summative to more formative, real‐time feedback mechanisms (Shute, 2008), in which lessons are crafted to make LP apparent.

The relation between decreased LP and increased likelihood of disengagement was only present for a subset of children. This is unlikely to indicate an inability to disengage depending on LP as a comparable ability has been identified much earlier in life (Poli et al., 2020) and was shared across the great majority of infants (Poli, Ghilardi, et al., 2023). Rather, it may indicate that when children engage with unknown environments and are left completely free to explore, multiple conflicting factors might influence their behavior (Liquin et al., 2021; Molinaro et al., 2023; Nussenbaum et al., 2020). On one side, LP leads them to improve their performance and reduce uncertainty. On the other side, they are attracted to familiar situations, possibly because they are under their control.

### Future directions

Compared to previous studies, the current work allows for a model‐based, trial‐by‐trial tracking of children's PEs and LP. In turn, this allows us to test more fine‐grained predictions about the unfolding of their exploratory behavior. Future studies should test whether findings obtained with a screen task extend to large‐scale search contexts (Pellicano et al., 2011) or free‐play contexts (Chu & Schulz, 2023; Le et al., 2021).

A further key question is whether the interindividual differences we observe depend on each child's state at the time of testing (e.g., whether they are tired, distractable, or comfortable in the lab environment at that specific moment) or whether there is a trait that accompanies their learning and exploration across development. In case the latter is true, these fundamental differences in how children explore might affect other important aspects of life, such as their interest in school, their curiosity, openness to experience, and ultimately academic and work achievements (Von Stumm et al., 2011). Future studies should systematically investigate what causes the emergence of distinct exploration profiles, and their relation to positive developmental outcomes (i.e., are some profiles more beneficial than others?).

Finally, future research should test this or similar tasks across cultures and at different ages (e.g., in school‐age children and adolescents) to determine the generalizability of our findings. This will also allow for a better understanding of the mechanisms underlying developmental change in curiosity‐driven exploration.

## CONCLUSIONS

This study sheds light on the principles that guide curiosity‐driven exploration in 4‐year‐old children. Our results show that children are not solely attracted to novel events but also preferentially explore activities from which they can learn the most. Furthermore, we found that individual differences in performance were related to children's engagement with the task. Children who performed better were also engaged depending on the LP they were making, while children who performed worse were also more focused on familiar activities. These findings extend the existing literature on infants and adults to provide new insights for understanding the developmental change in exploration behavior, possibly indicating some degree of continuity of its underlying mechanisms across the lifespan.

## AUTHOR CONTRIBUTIONS

F.P.: Conceptualization, investigation, data curation, methodology, software, formal analysis, writing—original draft, and visualization. M.M.: Conceptualization, supervision, and writing—review & editing. R.B.M.: Conceptualization, supervision, writing—review & editing, and funding acquisition. S.H.: Conceptualization, supervision, writing—review & editing, and funding acquisition.

## CONFLICT OF INTEREST STATEMENT

The authors declare that they have no competing interests.

## Supporting information


Data S1.

## Data Availability

Data, computational models, and statistical analyses scripts are available on OSF: https://osf.io/tsv6q/?view_only=199d7632237d433f9cb11a393eeca9f2. The analyses presented here were not preregistered.
