# Supplementary material for: Exploration in 4‐year‐old children is guided by learning progress and novelty
Source: Child Dev. 2024 Sep 2;96(1):192–202. doi: 10.1111/cdev.14158 (PMC11693834; doi:10.1111/cdev.14158)
Supplement: Supplementary file 1 — Data S1. [file CDEV-96-192-s001.docx]

Supporting Information

Exploration in 4-year-old children is guided by

learning progress and novelty

**Computational model**. On every trial, the model uses the incoming evidence (i.e., the location of the character) to update its expectations about the mean and the standard deviation of hidden probability distribution of the character (i.e., its pattern). The model can also detect change-points (i.e., when the mean of the distribution changes abruptly). Specifically, after sampling a new piece of evidence, the model computes the probability that a change-point *cp* occurred:

1. $p\left( cp|X_{t} \right)=\frac{{U(X}_{t}\left| 1,43 \right)H}{{U(X}_{t}\left| 1,43 \right)H+N(\hat{\mu}_{t},\hat{\sigma}_{t})(1-H)}$

where U and N indicate a uniform and a normal distribution, respectively. Hence, the hidden probability distribution of a given character is uniform over the entire space if a change-point has occurred, and it is normal distribution with mean $\hat{\mu}_{t}$ and standard deviation $\hat{\sigma}_{t}$ if a change-point has not occurred. $X$ is a vector containing all the evidence that has been observed and $X_{t}$ is the last piece of evidence that has been observed; H is a constant that specifies the average hazard rate of any given sequence, where the higher the hazard rate, the more frequently the mean changes. The integer 43 indicates the number of different locations along the hedge where the characters could hide.

The expected mean and standard deviation of the distribution are updated trial by trial, and both depend on whether a change-point has been estimated to occur or not. When estimating the mean, if a change-point occurred, only the last piece of evidence that has been observed should be considered:

1. $\hat{\mu}_{t}^{cp}=X_{t}$

Hence, all the evidence up until trial t-1 will be disregarded. Conversely, if a change-point has not occurred, also older evidence is considered:

1. $\hat{\mu}_{t}^{\neg cp}=\frac{X_{t}+\hat{r}_{t}\hat{\mu}_{t-1}}{\hat{r}_{t}+1}$

where the expected run length $\hat{r}_{t}$ indicates the expected number of consecutive trials (i.e., a run) since the last change-point. In words, equation (3) integrates the evidence acquired in the last trial with the expectations that the model had up until the previous point in time, weighting them for the time that passed from the last change point. The final estimate of the mean keeps into account both the possibility that a change point occurred and the possibility that it did not, weighting them for the probability that a change-point has occurred:

1. $\hat{\mu}_{t}=\hat{\mu}_{t}^{cp}p\left( cp|X_{t} \right)+\hat{\mu}_{t}^{\neg cp}(1-p\left( cp|X_{t} \right))$

Since inferring the current mean is a weighted integration of past expectations and new evidence, it can be re-written as a reinforcement learning algorithm (often called Rescorla-Wagner rule):

1. $\hat{\mu}_{t}=\hat{\mu}_{t}+\alpha_{t}\left( X_{t}-\hat{\mu}_{t} \right)$

where $\alpha_{t}$ is the learning rate that regulates the extent to which the difference between previous expectations and new evidence (i.e., the prediction error) will change future expectations. The learning rate is computed as follows:

1. $\alpha_{t}=\frac{1+p\left( cp|X_{t} \right)\hat{r}_{t}}{\hat{r}_{t}+1}$

In words, whether the prediction error will be considered informative depends on the probability that the current evidence indicates a change point, weighted by the expected run length $\hat{r}_{t}$.

Together with the estimation of the mean of the distribution, the model also estimates its standard deviation. The expected standard deviation of the probability distribution is updated adjusting the estimate of the previous trial given the new estimated mean, taking into account the hazard rate and the change-point probability:

1. ${SD}_{t+1}^{2}={SD}_{t}^{2}+\left( \frac{\hat{r}_{t}(X_{t}-\hat{\mu}_{t})}{\hat{r}_{t}+1}-{SD}_{t}^{2} \right)kH(1-p\left( cp|X_{t} \right))$

where k is a scaling constant. Hence, by keeping track of the change-point probability, we can estimate and update trial by trial the mean and the standard deviation of the hidden probability distributions of the three characters. From these estimates, we can compute the current and the expected learning progress. The current learning progress ${LP}_{t}$ is computed as the difference in prediction error from the previous and the current trial:

1. ${LP}_{t}=(X_{t-1}-\hat{\mu}_{t-1}) -(X_{t}-\hat{\mu}_{t})$

This indicates how much the model has changed its prediction error during the last timepoint. Hence, it is a measure of how much the model has improved (or gotten worse). To estimate the expected learning progress ${LP}_{expected(t+1)}$, a similar computation is carried out, but instead of using the prediction error from the past, the current prediction error is subtracted by the expected prediction error:

1. ${LP}_{expected(t+1)}=\left( X_{t}-\hat{\mu}_{t} \right)-{PE}_{expected(t+1)}$

where ${PE}_{expected(t+1)}$ is the expected prediction error that the model estimates to make in the future. ${PE}_{expected(t+1)}$ is estimated with a reinforcement learning algorithm:

1. ${PE}_{expected(t+1)}={PE}_{expected(t)}+\alpha_{t}(\left( X_{t}-\hat{\mu}_{t} \right)-{PE}_{expected(t)})$

It is important to note that equation (5) is a reinforcement learning algorithm that was updating the expected mean, while this is a second reinforcement learning algorithm that relies on the first to update the expected prediction error. Hence, a hierarchical structure emerges, where the expected mean is estimated at the first layer of the reinforcement learning algorithm, and the prediction error is estimated at the second layer. Following Wittman and colleagues (2016), we used the same learning rate $\alpha_{t}$ for both layers and, since the number of trials for each participant was low, individual-level estimates of $\alpha_{t}$ were averaged across participants.

Finally, the more one is exposed to a given environment, the less novel it is. We quantified perceptual novelty (N) as a parametric function of negative overall exposure to a given character:

1. $N=f\left( -t \right)$

where t indicates the overall number of trials a given character has been observed. In previous research (Poli et al., 2022), we show that we can successfully recover the learning rate $\alpha$ given the number of participants and trials in our data.

We used the variables $PE$, $LP$, ${PE}_{expected}$, ${LP}_{expected}$, $N$, to predict when the participants switched environments and what environment they decided to sample next using generalized mixed-effects models.
